# Euglycemic Diabetic Ketoacidosis Precipitated by a Keto Diet: Importance of Dietary History in Diagnosis

**DOI:** 10.7759/cureus.10199

**Published:** 2020-09-02

**Authors:** Safia Shaikh, Mohamed MG Mohamed, Abdul Mujeeb, Faizan Shaikh, Benjamin Harris

**Affiliations:** 1 Internal Medicine, SSM Health St. Mary's Hospital, St. Louis, USA; 2 Urology, Jinnah Postgraduate Medical Centre, Karachi, PAK; 3 Internal Medicine, Western Michigan University, Kalamazoo, USA

**Keywords:** euglycemic, diabetic ketoacidosis, dietary history, ketogenic, keto diet, diabetic diet

## Abstract

Diabetic ketoacidosis (DKA) is one of the serious complications of diabetes, especially type 1. It is defined by the triad of hyperglycemia (>250 mg/dL [>13.9 mmol/L]), high anion-gap metabolic acidosis, and increased plasma ketones. Euglycemic ketoacidosis is characterized by DKA without hyperglycemia. We present a rare case of a 28-year-old type 1 diabetic male, presenting with abdominal pain, fatigue, and dizziness after one week of starting a keto diet. He was diagnosed with euglycemic DKA, managed with DKA protocol and given detailed dietary counselling to avoid the keto diet in future.

## Introduction

A keto diet is a strict low-carbohydrate and high-fat diet. It has recently been very famous in the United States and globally due to its fast weight loss benefits [1,2]. The reduction in carbohydrates enables the body to rely on fat as a predominant energy source leading to a state of ketosis. Due to the increased publicity of faster weight loss, it has been one of the top diets. As there is limited data, long-term effects are not well established, but life-threatening complications of ketoacidosis have been noted [3].

## Case presentation

A 28-year-old type 1 diabetic male presented with three episodes of vomiting and vague abdominal pain associated with fatigue, weakness, and dizziness. He denied fever, chest pain, shortness of breath. He was diagnosed with type 1 diabetes at the age of 10 years and since then had been on Insulin. He was not compliant to measuring blood glucose regularly and relied on symptoms including diaphoresis and blurry vision to know if his blood glucose was high or low. For the last couple of years, he has been taking neutral protamine Hagedorn (NPH) insulin 60 units BID inconsistently. He was previously not compliant to the diabetic diet; however, one week before presentation, he started a strict keto diet plan, restricting carbohydrates to 15-20 g/day to better control his diabetes.

On exam, he was alert and oriented X4, hypovolemic with blood pressure of 80/50 mmHg, heart rate in the 130s, respiratory rate of 19, oxygen saturation 97% on room air, and mild abdominal tenderness was appreciated. Blood work showed glucose 109 mg/dL (normal range: 80-140 mg/dL), hemoglobin 18.2 g/dL (normal range: 12.0-17.6 g/dL), hematocrit 52.1% (normal range: 35.2%-51.7%), white blood cell (WBC) count 8.6 x 10^9^/L (normal range: 4.4-10.7 x 10^9^/L), platelets 329 x 10^9^/L (normal range: 153-416 x 10^9^/L), sodium 133 mmol/L (normal range: 135-145 mmol/L), potassium 4.6 mmol/L (normal range: 3.5-4.5 mmol/L), magnesium 1.8 mg/dL (normal range: 1.6-2.6 mg/dL), phosphorus 3.0 mg/dL (normal range: 2.3-4.7 mg/dL), chloride 102 mmol/L (normal range: 98-107 mmol/L), bicarbonate 14 mmol/L (normal range: 23-32 mmol/L), anion gap 19 mmol/L (normal range: 8-14 mmol/L), blood urea nitrogen (BUN) 19 mg/dL (normal range: 8.9-20.6 mg/dL), creatinine 2.04 mg/dL (normal range: 0.75-1.25 mg/dL), troponin I <0.010 ng/mL (normal range: <0.035 ng/mL), lipase 12 U/L (normal range: 8-78 U/L), beta-hydroxybutyrate 4.6 mmol/L (normal range: <0.6 mmol/L), and hemoglobin A1c 10.3% (normal range: 4.2%-5.3%). Venous blood gas on room air showed pH 7.26 (normal range: 7.31-7.41) and partial pressure of carbon dioxide (pCO_2_) 30 mmHg (normal range: 41-51 mmHg); chest X-ray showed normal lungs.

**Figure 1:**
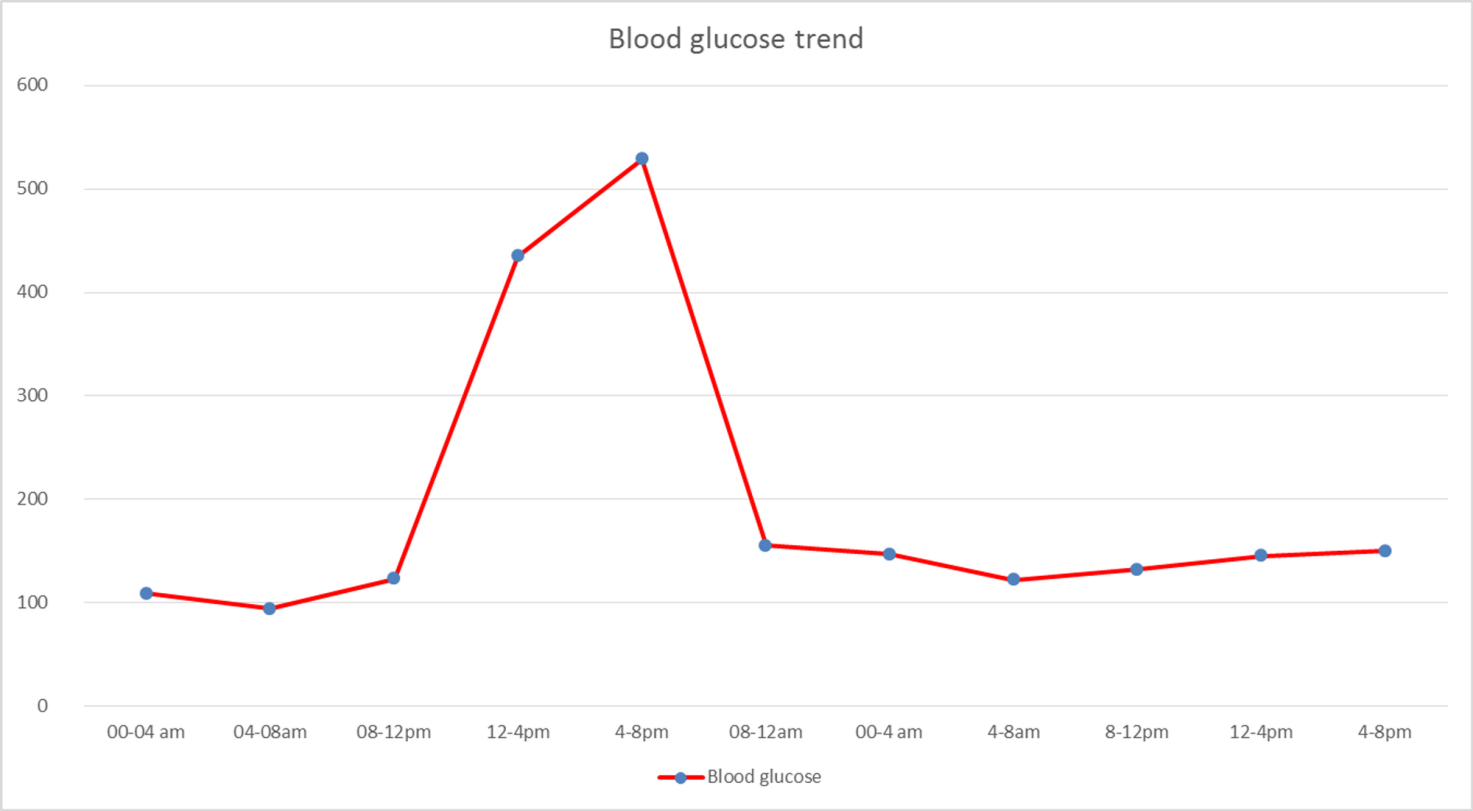
Blood glucose trend of our patient during the course of hospitalization

He was diagnosed with euglycemic ketoacidosis and managed with aggressive hydration and electrolyte repletion as per the DKA protocol. Insulin was cautiously given due to normoglycemia. The next day, his symptoms improved; labs showed the anion gap normalized to 8 mmol/L and bicarbonate improved to 21 mmol/L. His insulin infusion was overlapped and later switched to subcutaneous insulin. He was then given diabetic diet with consistent carbs and his blood glucose peaked to 500s (as shown in Figure 1). His diet was extensively reviewed, and he seemed to have an inconsistent dietary pattern. Nutritionist consultant and diabetic education were provided. He was advised to eat three meals a day and insulin was adjusted as per his body requirements. On the third day, his blood glucose was in an optimum range; he was discharged on insulin glargine and advised follow-up by an outpatient endocrinologist.

## Discussion

This case is an interesting example of dietary history providing clues to the diagnosis. The keto diet is a strict low-carbohydrate, high-fat, and adequate-protein diet. It has been previously used for the treatment of seizure disorders, neurodegenerative disorders, and has more recently become mainstream as a method for weight loss [1,4]. The aim of this diet is to enter a state of nutritional ketosis, deriving energy from fat burn-down, in the form of ketones. Short-term benefits have been postulated by faster weight loss, improved cognitive functioning, appetite suppression, and improved eating behaviour and metabolic profile [2]. However, the long-term benefits have been largely unexplored [5]. Though nutritional ketosis has been linked to a neuroprotective effect in Alzheimer's, the ketoacidosis can be neurotoxic to brain [3].

The high-fat and low-protein diet is unlikely to be cardioprotective as it may cause an increase in low-density lipoprotein and triglycerides [1]. Though increased carbohydrates have always been deemed for worst outcomes, further data is needed to clarify the risk versus benefit of a low-carbohydrate diet causing short-term weight loss with dyslipidemia in diabetic populations.

Diabetic ketoacidosis (DKA) is a medical emergency. It occurs due to a combination of insulin deficiency and activation of counterregulatory hormones (glucagon, cortisol, growth hormones, and catecholamines) causing hyperglycemia, glycosuria, and thus dehydration. DKA also causes release of fatty acids from adipose tissue and liver, resulting in their oxidative phosphorylation to ketone bodies resulting in ketonemia and metabolic acidosis [6]. Euglycemic DKA is equally life threatening and unlike hyperglycemic DKA can be diagnostically challenging. Peters et al. described the cases when providers failed to identify DKA due to normal glucose and workup was done for alternate causes [7]. Dizon et al. reported the delay in appropriate diagnosis in greater than 50% of cases [8]. Therefore, a high index of suspicion is required for diabetic patients presenting with acute metabolic acidosis despite optimum blood glucose.

A strong association has been described between euglycemic ketoacidosis with sodium-glucose co-transporter-2 (SGLT2) inhibitors (canagliflozin, dapagliflozin, and empagliflozin) due to glycosuria that causes increased glucagon level, lipolysis, and ketone production [7,9]. Also, starvation, alcoholism, change in insulin dosing, and infections are well-known causes of ketoacidosis and have been associated factors in patients taking a keto diet [1]. Our patient is a rare presentation as he did not change his eating patterns, denied starvation, with no alcohol intake or recent change in the insulin dosage while starting the keto diet one week before presenting to the emergency department. This emphasizes the fact that the keto diet itself can lead to dangerous consequences of ketoacidosis in diabetics without any other triggers.

## Conclusions

Diet and exercise patterns are of paramount importance in determining optimum blood glucose control in diabetics. Keto diet might have been related to health benefits by causing faster weight loss in the obese population including type 2 diabetics; however, nutritional ketosis may lead to potentially life-threatening euglycemic ketoacidosis. It can be diagnostically challenging in diabetics due to normal glucose levels, so strong clinical suspicion should be maintained. Once diagnosed, the DKA protocol should be initiated to maintain blood glucose and prevent further ketosis. Detailed nutritional and diabetic education cannot be overemphasized.
